# Hyperfine Interactions in the NV-^13^C Quantum Registers in Diamond Grown from the Azaadamantane Seed

**DOI:** 10.3390/nano11051303

**Published:** 2021-05-14

**Authors:** Alexander P. Nizovtsev, Aliaksandr L. Pushkarchuk, Sergei Ya. Kilin, Nikolai I. Kargin, Alexander S. Gusev, Marina O. Smirnova, Fedor Jelezko

**Affiliations:** 1 National Research Nuclear University “MEPhI”, 115409 Moscow, Russia; alexp51@bk.ru (A.L.P.); NIKargin@mephi.ru (N.I.K.); ASGusev@mephi.ru (A.S.G.); mosmirnova@yandex.ru (M.O.S.); 2 Institute of Physics, Nat. Acad. Sci. of Belarus, 220072 Minsk, Belarus; sergei_kilin@yahoo.com; 3 Institute for Quantum Optics, Ulm University, 89069 Ulm, Germany; fedor.jelezko@uni-ulm.de

**Keywords:** nitrogen-vacancy (NV) center, seeded diamond growth, azaadamantane, isotopic ^13^C nuclear spin, hyperfine interaction, density functional theory

## Abstract

Nanostructured diamonds hosting optically active paramagnetic color centers (NV, SiV, GeV, etc.) and hyperfine-coupled with them quantum memory ^13^C nuclear spins situated in diamond lattice are currently of great interest to implement emerging quantum technologies (quantum information processing, quantum sensing and metrology). Current methods of creation such as electronic-nuclear spin systems are inherently probabilistic with respect to mutual location of color center electronic spin and ^13^C nuclear spins. A new bottom-up approach to fabricate such systems is to synthesize first chemically appropriate diamond-like organic molecules containing desired isotopic constituents in definite positions and then use them as a seed for diamond growth to produce macroscopic diamonds, subsequently creating vacancy-related color centers in them. In particular, diamonds incorporating coupled NV-^13^C spin systems (quantum registers) with specific mutual arrangements of NV and ^13^C can be obtained from anisotopic azaadamantane molecule. Here we predict the characteristics of hyperfine interactions (*hfi*) for the NV-^13^C systems in diamonds grown from various isotopically substituted azaadamantane molecules differing in ^13^C position in the seed, as well as the orientation of the NV center in the post-obtained diamond. We used the spatial and *hfi* data simulated earlier for the H-terminated cluster C_510_[NV]^-^H_252_. The data obtained can be used to identify (and correlate with the seed used) the specific NV-^13^C spin system by measuring, e.g., the *hfi*-induced splitting of the m_S_ = ±1 sublevels of the NV center in optically detected magnetic resonance (ODMR) spectra being characteristic for various NV-^13^C systems.

## 1. Introduction

Hybrid spin systems consisting of the electronic spin of single paramagnetic color centers (PCC) in diamond and neighbor nuclear spins are now widely used to implement numerous applications ranging from quantum technologies to biological sciences (see, e.g., reviews [1,2,3,4,5,6,7,8]). In these systems, the nuclear spins with their long coherence times serve as quantum memories accessed via the more easily controllable electronic spin of the PCC which, in turn, can be initialized and readout using optical photons. The well-known representative of such systems is the “nitrogen-vacancy” (NV) center in diamond having electron spin S = 1 coupled by hyperfine interactions (*hfi*) to I = 1/2 spins of ^13^C atoms that usually are distributed randomly in diamond lattice substituting spineless ^12^C atoms with the 1.1% probability under natural conditions. As is well-known [1,2], the NV center possesses unique interconnected optical and spin properties, allowing optical initialization and high-fidelity readout of its electronic spin state, and can be used to control ^13^C nuclear spins [3]. Currently, the techniques for coherent manipulation of the spin state of NV-^13^C complexes to perform quantum algorithms or single-shot readout of both nuclear and electronic spins, as well as realize entanglement-based sensitivity enhancement of a single-spin quantum magnetometry and metrology, are well established [1,2,3,4,5,8]. In particular, usage of dynamical decoupling methods [9] or high-resolution ESR spectroscopy [10] allows experimental observation the NV-^13^C spin systems wherein the ^13^C atom is disposed rather far from the NV center. It should also be noted that during recent time the technology of growing artificial diamonds has been significantly improved [11,12] allowing, in particular, synthesize samples of isotopically pure diamond with a low content of ^13^C atoms, as well as diamond samples in which ^13^C atoms are distributed in the form of a thin layer [13]. NV centers in such samples are created either during crystal growth or are then introduced via ion implantation with subsequent annealing. In the context of this article, the main disadvantage of all these methods is that all of them are inherently probabilistic with respect to mutual location of the color center electronic spin and the ^13^C nuclear spins.

A recently new bottom-up approach to fabricate such systems was suggested [14,15,16] based on the idea to synthesize first chemically appropriate organic molecules containing desired constituents (including nuclear spins of ^13^C) in definite positions, and then use them as a seed for high pressure, high temperature (HPHT) growth to produce macroscopic diamonds. Among organic molecules, the most natural seed for growing diamond are diamondoids [17,18,19], which initially have a diamond structure. The smallest possible diamondoid is adamantine C_10_H_16_, containing ten carbon atoms arranged as a single diamond cage, terminated by 16 hydrogen atoms. Larger diamondoids contain multiple H-terminated adamantanelike diamond cages. The chemistry of diamondoids has been studied very extensively (see, e.g., [17,18,19,20,21,22]) and numerous derivatives may be formed chemically either by changing the surface termination and/or substituting carbon atoms with other elements to get the heterodiamondoids [23,24], in particular the azaadamantane C_9_H_15_N [25], which itself is obtained from ordinary adamantine by replacing one of the carbon atoms with nitrogen atom, and which is of primary interest here as the seed for diamond growth.

To date, quite a lot of successful experimental implementations of the HPHT synthesis of diamond from diamondoids and their various derivatives have been performed [16,26,27,28,29,30]. Formation of nanodiamonds in these works was confirmed using X-ray diffraction, Raman spectroscopy, scanning (SEM) and transmission (TEM) electron microscopy. Although these studies demonstrated the promise of using diamondoids in seeded diamond growth, one of the central challenges in them was the thermal decomposition of the seed at high P-T conditions of experiments. This problem was overcome recently in [31], where a low-temperature (T = 4000, P = 10 GPa) slow (24 h) synthesis of high-quality nanodiamonds was implemented in a laser-heated diamond anvil cell [32] using 2-azaadamantane as a seed and some other less thermally stable hydrocarbon molecules for crystal growth. After irradiation with electrons and subsequent annealing, the resulting nanodiamonds revealed the presence of bright NV centers for which high-contrast spectra of optically detectable magnetic resonance (ODMR) were obtained [31]. If, according to the original idea of the work [14], instead of the ordinary azaadamantane, one uses isotopically substituted azaadamantane, in which the usual ^12^C carbon atoms at particular locations are replaced by ^13^C atoms, then the method of the work [31] could be used to synthesize nanodiamonds containing the compound-systems NV-^13^C having chemically determined mutual arrangements of NV and ^13^C. ODMR spectra or other high-resolution ESR methods can be used to study such coupled spin systems and to elucidate many unknown details of diamond growth from such seeds. Obviously, for these purposes it is necessary to know in detail the characteristics of hyperfine interactions (*hfi*) in such systems. It is the aim of this work to predict such *hfi* characteristics for various isotopically substituted azaadamantanes seeds using for this purpose previously obtained [33] data on the spatial and *hfi* characteristics for various NV-^13^C systems in the cluster C_510_[NV]-H_252_ which mimics the nanodiamond grown from the seeds. First preliminary results reporting such modeling were recently published in [34].

## 2. Materials and Methods

**Figure 1:**
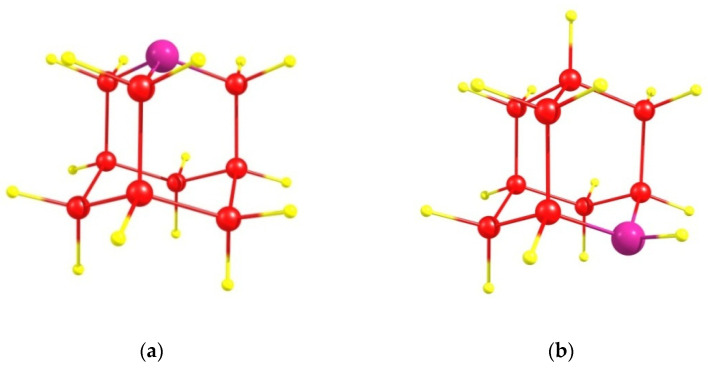
Azaadamantane molecules: (**a**) 1-azaadamantane molecule (4 equivalent positions for the N atom) and (**b**) 2-azaadamantane molecule (6 equivalent positions). Nitrogen atom is shown in purple, carbon atoms–in red and terminating hydrogen atoms–in yellow.

We consider the azaadamantane molecules C_9_H_15_N (Figure 1a,b) as a seed for the growth of diamond doped with NV centers. As is well known [24,25], in adamantane there are four equivalent positions (usually called the bridgehead positions) for the substituted nitrogen atom in which it is bonded to three nearest carbon atoms (1-azaadamanrane, see Figure 1a), as well as 6 other equivalent positions (bridge positions) in which the N atom is bonded to only two nearest carbon atoms (2-azaadamantane, see Figure 1b). Both of these compounds can be synthesized [23,25] with the second one being more common due to more reactive bridge position in the adamantine skeleton. Using respective isotopic ingredients during synthesizing, the ordinary spinless ^12^C atoms in azaadamantine can be replaced by an isotopic ^13^C atom having nuclear spin I = 1/2, thus creating various isotopic seeds differing in the mutual arrangement of N and ^13^C atoms in the azaadamantane molecule. As a result, azaadamantane has 9 possible positions for ^13^C with some of them being equivalent. Additionally, surface terminating H atoms can be changed chemically to, e.g., methyl group –CH_3_, containing isotopic ^13^C atoms. So, a lot of various seeds containing isotopic ^13^C atoms located in different positions with respect to the N atom can be created chemically on the basis of azaadamantane.

**Figure 2:**
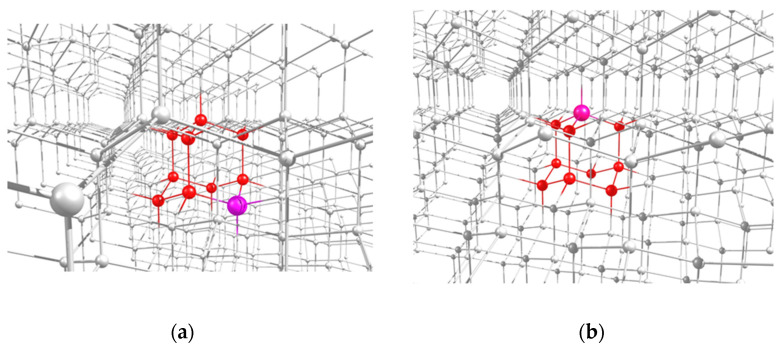

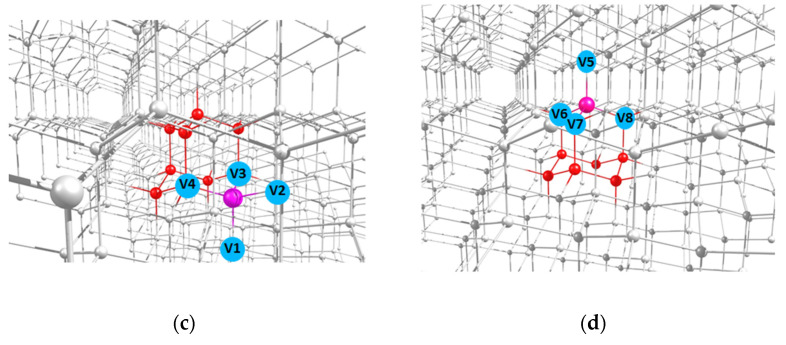
Highlighted locations of the 2-azaadamantane (**a**) and 1-azaadamantane (**b**) seedsin the lattice of diamond nanocrystals grown from them and (**c**,**d**) possible positions of the vacancies V1–V4 and V5–V8 in the NV1–NV8 centers formed in the nanocrystals obtained from the two above seeds after electron irradiation and annealing.

Here, following earlier work [31], we restrict ourselves to considering the 2- and 1-azaadamantane molecules as a seed for diamond growth, and will study their various isotopic derivatives containing the ^13^C nuclear spin in different positions. An example of the location of the both molecular seeds in the lattice of a grown nanodiamond are shown in Figure 2a,c. After completion of diamond growth from the seed and subsequent processing of the resulting nanodiamonds, vacancies can be created in them by electron irradiation, which, during subsequent annealing, migrate over the grown nanocrystal, eventually forming the NV center with the nitrogen atom being a part of the seed. In accordance with four possible positions of a vacancy in a diamond crystal relative to the nitrogen atom fixed in the lattice, the formed NV center can have four possible orientations, as is shown in the Figure 2c,d by light-blue circles V1–V8 for 2- and 1-azaadamantanes, respectively. Below we will refer to the corresponding NV centers as NV1–NV8 centers.

Our aim here is to present the characteristics of hyperfine interaction (*hfi*) of the electron spin associated with NV centers with the nuclear spins of isotopic ^13^C atoms belonging to the seed. Obviously, they will depend on the location of the ^13^C and orientation of the NV centers. Therefore, when predicting the *hfi* characteristics for various NV-^13^C spin systems in diamond grown from isotopically modified azaadamantane it is necessary to consider all possible cases V1–V8 of the vacancy location relative to the nitrogen atom in the seed. It can be done using quantum chemistry simulation of H-terminated diamond cluster hosting NV center. Here we will use for the purpose the C_510_[NV]-H_252_ cluster for which we previously [33] found spatial coordinates of all possible locations of the ^13^C atom C(j) (j = 1 ÷ 510) with respect to the NV center and calculated full *hfi* matrices A_KL_ (K,L = X,Y,Z) for every possible position of the ^13^C nuclear spin at fixed orientation of the NV center located in the central part of the cluster (see [33] for details). In [33] we also used the calculated *hfi* matrices in the standard ground-state spin Hamiltonian of the NV center (see, e.g., Equation (1) in [33] or Equations (1) and (2) in [35]) to calculate numerically the signatures of *hfi* in optically detected magnetic resonance (ODMR) spectra of various ^14^NV-^13^C spin systems viz. the *hfi*-induced splitting Δ_0_(at zero external magnetic field) of the m_S_ = ±1 sublevels of the NV center the value of which is characteristic of the specific NV-^13^C system. Note that recently our predictions [33] got few direct experimental confirmations [36,37,38]. Here the data of [33] will be used to find the *hfi* characteristics as well as the *hfi*-induced splitting Δ_0_ for the spin systems NV-^13^C wherein the NV center has eight possible orientations NV1–NV8 and nuclear spin ^13^C is located in one of the nodes of the azaadamantane seeds. It will be done by rotating the cluster C_510_[NV]-H_252_ with the ChemCraft software package to obtain the desired relative configuration of the NV1–NV8 centers and various isotopic seeds. This makes it possible to identify the numbers j of the carbon atoms C(j) belonging to the seed in accordance with their numbering in the cluster of the article [33] and then using the Supplement to the article, one can find the *hfi* characteristics for this specific NV-^13^C(j) system obtained from respective seed as a result of diamond growth and creation a vacancy in one of the lattice site near the nitrogen atom. The results of such analysis are presented below in the Table 1 both for 2- and 1-azaadamantane seeds.

## 3. Results and Discussion

**Figure 3:**
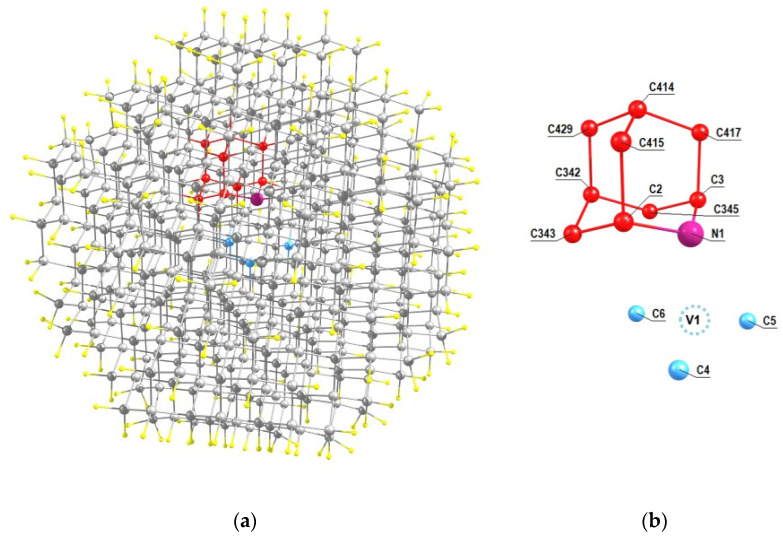
(**a**) Location of the 2-aza-adamantante seed in the C_510_[NV]-H_252_cluster of the work [33] at the NV1 orientation of the formed NV center and (**b**) the numeration of the carbon atoms of the seed (shown in red) in the cluster. Nearest neighbors of the vacancy V1 of the center are shown in light blue.

Basically, following the recent experimental work [31], we will discuss nanodiamonds with the NV center obtained from the seed, which was the isotopically substituted 2-azaadamantane, where some ^12^C carbon atoms were replaced by the ^13^C isotope. But in parallel we will also gave the results obtained by analogous way for the case of 1-azaadamantane seed. For simplicity, we will assume that the grown diamond nanocrystal contains no nuclear spins ^13^C other than those that are part of the seed. Accordingly, below we are presenting the data on the *hfi* characteristics for eight different orientations of the NV center relative to the seeds, in which each of the carbon atoms can be an isotopic ^13^C atom. For the case of the NV1 center (see Figure 2c), the relative position of this center and the seed in the respectively rotated C_510_[NV]-H_252_cluster is shown in Figure 3a. Figure 3b shows an enlarged view of this seed with indication of the numeration of the carbon atoms and the position of the vacancy relative tothe seed. In this case, there are 8 possible systems NV1-^13^C(j), in which the index j (j = 2, 3, 342, 343, 345, 415, 414, 417, 429) numbers the position of the ^13^C nuclear spin in the cluster C_510_[NV]-H_252_ of the work [33].

**Figure 4:**
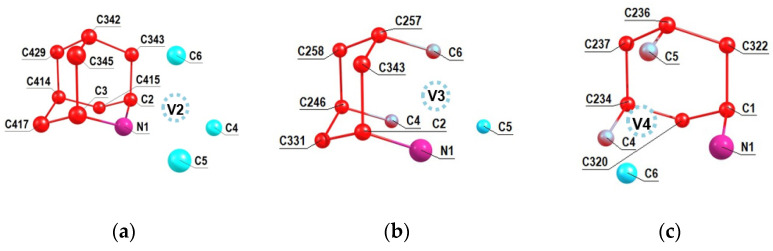
Numeration of carbon atoms in the 2-aza-adamantante seed in the C_510_[NV]-H_252_cluster at the NV2 (**a**), NV3 (**b**) and NV4 (**c**) orientations of the formed NV center with respect to the seed.

**Figure 5:**
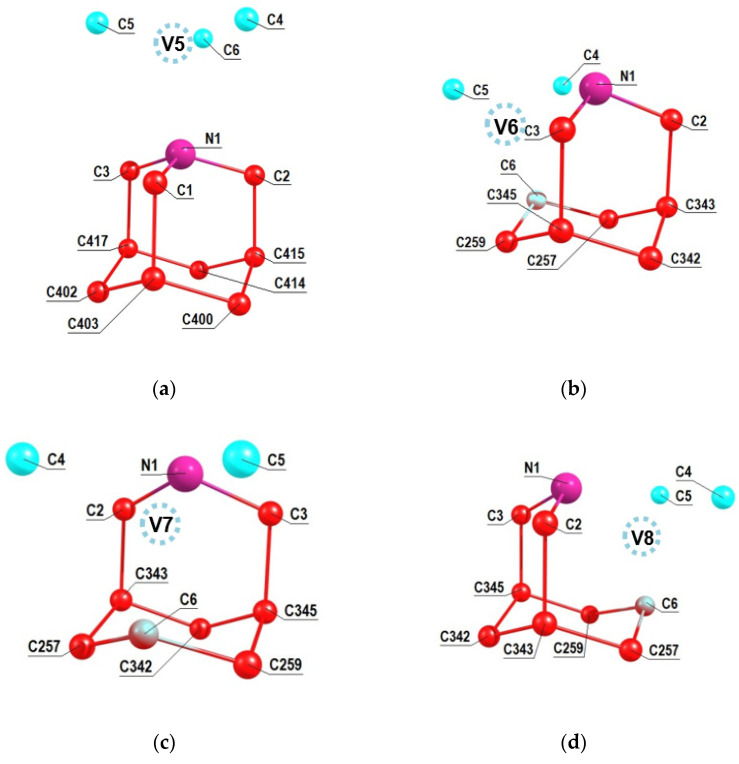
Numeration of carbon atoms in the 1-aza-adamantante seed in the C_510_[NV]-H_252_ cluster at the NV5 (**a**), NV6 (**b**), NV7 (**c**) and NV8 (**d**) orientations of the NV center with respect to the seed.

A similar analysis was performed for the other NV2, NV3 and NV4 orientations of the NV center relative to the 2-azaadamantant seed, the results of which are shown in Figure 4a–c. One can see from Figure 4b,c (see also Figure 2b) that in the cases of the NV3 and NV4 centers the vacancies V3 and V4 are formed by removing one of the carbon atoms that is part of the initial seed.Accordingly, in such cases, there can be eight NV-^13^C systems, and some of them can include ^13^C atoms, which are the nearest neighbors of the formed vacancy, which, as is well-known, are characterized by the strongest hyperfine interaction with the electron spin of the NV3 and NV4 centers. Analogous results shown in the Figure 5a–c have been obtained also in the case of 1-azaadamantane seed.

Using the Supplement to the article [33] with the account of the numbering of the carbon atoms C(j) in the seeds shown in Figure 3b, Figure 4 and Figure 5, it is easy to obtain the *hfi* characteristics for the corresponding systems NV-^13^C(j) which are presented below in Table 1, the first part of which corresponds to the systems obtained from the 2-azaadamantane seed and the secondone—from the 1-azaadamantane seed. In the first column of both parts in the Table 1 we show the system NV-^13^C(j) with indication of the vacancy position V1–V8 and the position number of the respective carbon ^13^C atom in the C_510_[NV]-H_252_ cluster. The second and third columns gave the respective values of the elements A_ZZ_ and $A_{\mathrm{nd}}=\sqrt{A_{\mathrm{ZX}}^{2}{+A}_{\mathrm{ZY}}^{2}}$ of the full *hfi* matrix A_KL_ (K,L = X,Y,Z) which was calculated in [33] in the coordinate system where Z axis was directed along the NV symmetry axis while the X and Y axes were taken arbitrary. Note that it is these matrix elements that determine the main experimentally observed manifestations of *hfi*, e.g., in the ODMR spectra (see [33] for details of this secular approximation), and the A_nd_ combination does not depend on the choice of the X and Y axes. In particular, if one choose the X axis so that the XZ plane passes through the considered ^13^C atom, thenone will have A_ZY_ = 0 [39]. The forth column of the Table 1 shows the numerically calculated values of the *hfi*-induced splitting Δ_0_ of the sublevels m_S_ = ±1 at zero external magnetic field, which can be measured experimentally and serve as the main parameter identifying the position of the ^13^C nuclear spin in the diamond lattice relative to the NV center.

The data presented in Table 1 clearly demonstrate essentially different *hfi* characteristics (and, respectively, different *hfi*-induced splitting of the zero-field ODMR lines) for different NV-^13^C systems, thus providing the way to identify the pair “seed-NV” and to determine position of the vacancy with respect to the N atom of the seed. To distinguish different NV-^13^C systems exhibiting approximately the same values of the *hfi*-induced splitting Δ_0_, it is possible to study the changes in the ODMR spectrum under the action of an external magnetic field.

Among the systems considered, the cases NV3-^13^C(4/6), NV4-^13^C(4/5) and NV6/7/8-^13^C(6), exhibiting largest *hfi*-induced splitting Δ_0_~134 MHz, correspond to the situation when the ^13^C nuclear spin is located in the position being closest to the vacancy, in which the electron spin of the NV center interacts most strongly with the nearest-neighbor nuclear spins. The next rather large splitting ~6.4 MHz takes place for other equivalent systems NV3-^13^C(246), NV3-^13^C(257), NV4-^13^C(234), NV4-^13^C(236) and NV6/7/8-^13^C257, NV6/7/8-^13^C(259), wherein the ^13^C atoms are the second neighbors ofrespective vacancies. For the other systems NV-^13^C, the *hfi*-induced splitting values are smaller but can be measured using well-known high-resolution ESR spectroscopy methods [3,10].

**Figure 6:**
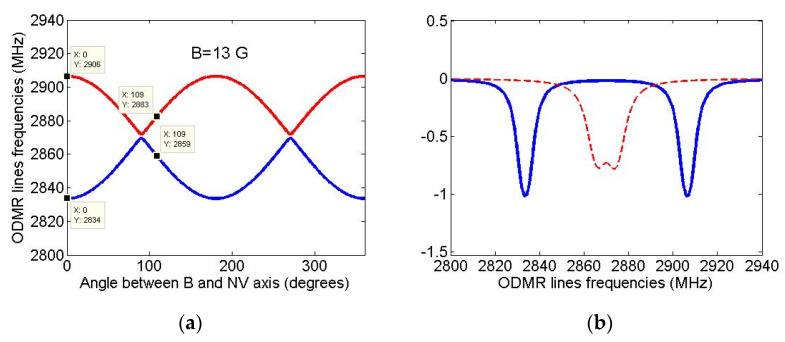
(**a**) Simulated dependence of ODMR line frequencies ω_+_ and ω_−_corresponding to the transitions m_S_ = +1→ m_S_ = 0 (red curve) and m_S_ = −1→ m_S_ = 0 (blue curve) on the polar angleθ, which determines the direction of the field B = 13 G relative to the center axis. It follows from the Figure 6a that the first NV center is aligned along the applied field, while the second one makes an angle of ~109°with this field. (**b**) Simulated ODMR spectra of the two NV centers studied in [31]. The solid blue curve corresponds to the NV center in the magnetic field B = 13 G being parallel to the axis of this center, the dashed red one—to the second NV center aligned at an angle of 109° to the applied magnetic field B = 4.7 G.

Since ODMR spectra can be used to control the diamond growth from certain isotopical seeds, it would be instructive to simulate the examples of ODMR spectra that can be observed for the above-described NV-^13^C systems. As was noted in the Introduction, recently the ODMR spectra of nanodiamonds grown from not modified isotopicallyazaadamantane were obtained in the experimental work [31] where Figure 2c shows the spectrum of nanodiamond containing two NV centers. The spectrum was taken in the presence of low external magnetic field B ~ 10 G of unknown direction and it consisted of four lines having rather large linewidths ~10–15 MHz. To interpret it, we first determined the orientation of these two NV centers relative to the direction of the external magnetic field B, calculating for an arbitrary NV center the dependence of the frequencies ω_+_ and ω_−_ of two ODMR lines corresponding to the transitions m_S_ = +1 ↔ m_S_ = 0 and m_S_ = −1 ↔ m_S_ = 0 in the magnetic field of a given strength B ~ 10 G on the polar angle θ that determines B field direction relative to the NV center axis (the dependence on the azimuthal angle φ is practically absent due to large fine splitting of m_S_ = ±1 and m_S_ = 0 states of the center [40]). The results of such simulation are shown in Figure 6a for a magnetic field of strength B = 13 G, at which the calculated values of the ODMR frequencies ω_+_ = 2906 MHz and ω_−_ = 2834 MHz at θ = 0° are in good agreement with the respective values ω_+_ ~ 2910 MHz and ω_−_ ~ 2835 MHz of the experimental work [31]. Since for the second NV center the experimental value of the m_S_ = +1 and m_S_ = −1 states splitting in the external magnetic field was much less (~10 MHz), we can assume that this NV center makes up an angle of ~109° (the tetrahedral angle of the diamond lattice) with the magnetic field B. However, with such an orientation of the second NV center, the predicted splitting ω_+_ – ω_−_ ~ 24 MHz (see Figure 6a) turns out to be significantly larger (at B = 13 G) than the experimental one. The reason may be a different location of the second NV center in this nanodiamond or the influence of localcharges in the vicinity of the second center [41]. Finally, we were able to describe simultaneously both experimental ODMR spectra of the work [31], assuming that they are differently oriented in the diamond lattice with the angle ~109° between their axes and that they are affected by magnetic fields of slightly different magnitudes. Using standard spin-Hamiltonian of the ground-state NV centers (see, e.g., Equations (1) and (2) in [35]) in respective magnetic fields we calculated first the δ-shaped ODMR spectra of both NV centers and then replaced them by Lorentzians of equal areas with a half-width of ~3 MHz. The results of such modeling are shown in Figure 6b, in which the solid bluecurve represents the ODMR spectrum of the first center in the field B = 13 G, being parallel to the axis of this center, and the dashed redone—the ODMR spectrum of the second center in the field B = 4.7 G, the axis of which makes an angle of 109° with the magnetic field direction. We note that the technique of seeded growth potentially allows use seed molecule with ^13^C located at desired position.

**Figure 7:**
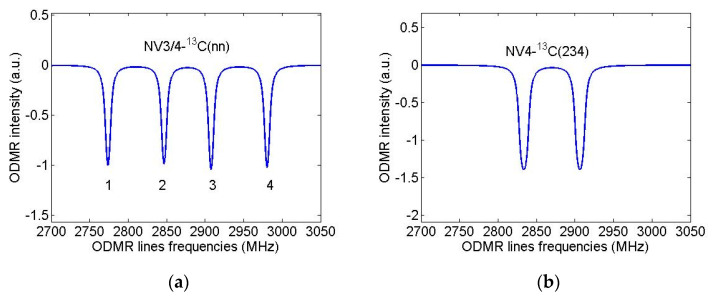
Simulated ODMR spectra of a single NV-C spin systems in nanodiamond grown from 2-azaadamantane with the subsequent formation of the vacancy V3 or V4. Magnetic field B = 13 G was assumed to be aligned along NV axis, ODMR linewidths (HWHM) were taken to be 3 MHz. (**a**) NV3-C(nn) spin system (Δ_0_ ~ 134 MHz), (**b**) NV4-C(234) spin system (Δ_0_ ~ 6.4 MHz).

**Figure 8:**
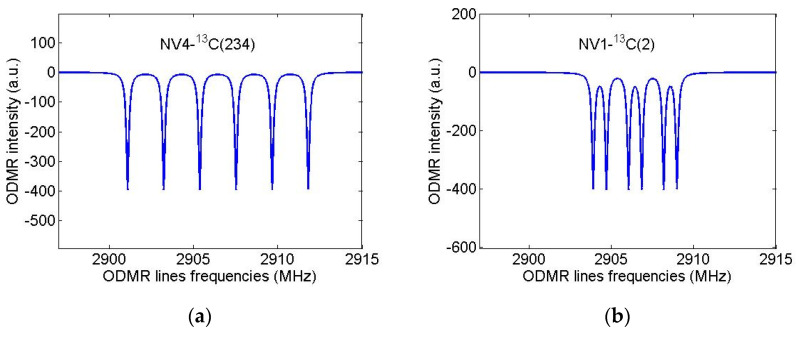
Simulated high-resolution ODMR spectra forthe NV4-^13^C(234) (Δ_0_ ~ 6429 kHz) (**a**) and NV1-^13^C(2) (Δ_0_ ~ 804 kHz) (**b**) spin systems in nanodiamonds grown from 2-azaadamantane. Magnetic field B = 13 G was assumed to be aligned along NV axis, ODMR linewidths were taken to be 0.1 MHz.

Let us now assume that the diamond nanocrystal studied in [31] contained only one first NV center and calculate again its ODMR spectrum in the field B = 13 G directed along the NV axis but will suppose that this nanocrystal was grown from isotopically substituted 2-azaadamantane seed.For the purpose, we again use the standard spin Hamiltonian of the NV center in magnetic field (see, e.g., Equation (1) in [35]), takingnow into account the *hfi* of the NV electron spin with the nuclear spin of the ^13^C atom, which is part of the seed.According to Table 1, in the case of sufficiently wide ODMR lines of [31], only systems NV3-^13^C(nn) and NV4-^13^C(nn) (nn = 4/5/6 is the number of the nearest neighbor of the vacancies V3 and V4) can be identified with certainty from such spectra, since for them the *hfi*-induced splitting significantly exceeds the ODMR line width. The result of modeling the ODMR spectrum for the NV3/4-^13^C(nn) system is shown in Figure 7a where instead of two lines in the spectrum of Figure 6b there are four lines with the line pairs 3,1 and 4,2 are due to the *hfi*-induced spitting Δ_0_ ~ 134 MHz. On the contrary, Figure 7b shows that even for the NV4-^13^C(234) spin system (Δ_0_ ~ 6.4 MHz) the *hfi* structure is not resolved in the ODMR spectrum at such a low resolution. Therefore, it is obvious, that the identification of systems in diamond nanocrystals grown from other isotopic seeds of 2-azaadamantane requires ODMR spectra having much higher spectral resolution to readout the hyperfine structure of ODMR lines. To illustrate these opportunities, we have simulated high-resolution ODMR spectra for the systems NV4-^13^C(234) and NV1-^13^C(2), shown in Figure 8a,b, respectively. Both spectra were calculated at a small half-width Γ = 100 kHz of the Lorentzians, simulating the ODMR lines broadening.

It can be seen that the spectra of Figure 8 demonstrate a well-resolved hyperfine structure, resulting from the hyperfine interaction of the NV electron spin with both the nuclear spin I = 1 of the nitrogen ^14^N atom of the seed and with the nuclear spin I = 1/2 of the corresponding ^13^C atom of the seed. As is well-known (see, e.g., [3,10]), *hfi* with ^14^N leads to the ODMR line splitting into three components spaced from each other by about 3 MHz. Additional interaction with the ^13^C nuclear spin doubles the number of lines in the ODMR spectrum. In the case of the system NV4-^13^C(234), the first interaction turns out to be less than the second one, while in the case of the system NV1-^13^C(2) the opposite situation takes place. Having the *hfi* data presented in Table 1, one can simulate similar high-resolution ODMR spectra for an arbitrary system NV-^13^C and, by comparing them with the experimentally obtained spectra, identify the seed, from which this nanocrystal with a NV center was grown. Note that ODMR spectra of ultrasmall nanodiamonds are often broadened by short coherence time associated with surface. Hence the experimentaldetectionof hyperfine coupling using high resolution ODMR spectroscopy requires improvement in nanodiamond synthesis.

## 4. Conclusions

In conclusion, we present the quantum chemical analysis of hyperfine interactions in macroscopic diamond nanocrystals obtained using their seeded growth from various isotopically substituted azaadamantane molecules. By combininghigh-resolution optically detected magnetic resonance spectroscopyand hyperfine characteristics presented in this work, one can identify the type of the seed from which the corresponding nanodiamond hosting the NV center was obtained. Hence, our modelcan serve as important fingerprint for experiments characterizing success of seeded nanodimaond synthesis. Similar data can be obtained for other isotopical heterodiamondoids, in particular, diadamantanes, triadamantanes, etc. Note that silicon or germanium atoms can be chemically substituted for carbon atoms in adamantanes (see, e.g., [6,16,42]), and it will be interesting to extend our studies to silicon-vacancy or germanium-vacancycolor centers in the future.

## Figures and Tables

**Table 1:** Predicted values of the *hfi* parameters A_ZZ_ and $A_{\mathrm{nd}}=\sqrt{A_{\mathrm{ZX}}^{2}{+A}_{\mathrm{ZY}}^{2}}$ and values of the *hfi*-induced splitting Δ_0_ of the m_S_ = ±1 sublevels for various NV-^13^C systems in diamond grown from isotopic 2-azaadamantane and from 1-azaadamantane at four different positions of the vacancy in the formed NV centers.

Name of Seed	Spin System in the C_510_[NV]-H_252_ Cluster	A_ZZ_ (kHz)	A_nd_ (kHz)	Δ_0_ (kHz)
2-azaadamantabe seed				
	NV1-C(2)	577	560	804
	NV1-C(3)	566	559	796
	NV1-C(342)	−224	396	455
	NV1-C(343)	3591	742	3666
	NV1-C(345)	3570	741	3645
	NV1-C(414)	152	93	178
	NV1-C(415)	345	150	376
	NV1-C(417)	341	150	373
	NV1-C(429)	641	169	663
	NV3-C(2)	577	560	803.8
	NV3-C(4)	136,870	19,877	134,100
	NV3-C(6)	136,810	19,767	134,020
	NV3-C(246)	−6339	933	6411
	NV3-C(257)	−6334	936	6407
	NV3-C(258)	968	121	974
	NV3-C(331)	3581	744	3656
	NV3-C(343)	3591	742	3666
	NV4-C(1)	560	560	792
	NV4-C(4)	136,870	19,877	134,100
	NV4-C(5)	136,520	19,964	133,780
	NV4-C(234)	−6357	933	6429
	NV4-C(236)	−6370	932	6442
	NV4-C(237)	1020	127	1026
	NV4-C(320)	3561	744	3637
	NV4-C(322)	3590	744	3665
1-azaadamantane seed				
	NV5-C(1)	560	560	792
	NV5-C(2)	577	560	804
	NV5-C(3)	566	559	795
	NV5-C(400)	153	93	179
	NV5-C(402)	153	93	179
	NV5-C(403)	338	151	370
	NV5-C(414)	152	93	178
	NV5-C(415)	345	150	376
	NV5-C(417)	341	150	373
	NV6-C(2)	577	560	804
	NV6-C(3)	56	559	795
	NV6-C(6)	136,810	19,767	134,020
	NV6-C(257)	−6334	936	6407
	NV6-C(259)	−6338	938	6411
	NV6-C(342)	−224	396	455
	NV6-C(343)	3591	742	3666
	NV6-C(345)	3570	741	3645
	NV7-C(2)	577	560	804
	NV7-C(3)	566	559	795
	NV7-C(6)	136,810	19,767	134,020
	NV7-C(257)	−6334	936	6407
	NV7-C(259)	−6338	938	6411
	NV7-C(342)	−224	396	455
	NV7-C(343)	3591	742	3666
	NV7-C(345)	3570	741	3645
	NV8-C(2)	577	560	804
	NV8-C(3)	566	559	795
	NV8-C(6)	136,810	19,767	134,020
	NV8-C(257)	−6334	936	6407
	NV8-C(259)	−6338	938	6411
	NV8-C(342)	−224	396	455
	NV8-C(343)	3591	742	3666
	NV8-C(345)	3570	741	3645
